# Healthcare cost of type 1 diabetes mellitus in new-onset children in a hospital compared to an outpatient setting

**DOI:** 10.1186/1471-2431-13-55

**Published:** 2013-04-15

**Authors:** Christopher F Jasinski, Rosa Rodriguez-Monguio, Ksenia Tonyushkina, Holley Allen

**Affiliations:** 1 School of Public Health and Health Sciences, University of Massachusetts, Amherst, 715 N. Pleasant Street, Amherst, MA, 01003, USA; 2 Department of Pediatrics, Tufts University School of Medicine, Baystate Health, 759 Chestnut Street, Springfield, MA, 01199, USA

**Keywords:** Diabetes, Healthcare costs, Healthcare utilization, Healthcare delivery, Pediatric population, Economic evaluation

## Abstract

**Background:**

Type 1 diabetes is among the most prevalent chronic childhood diseases in the US. Initial type 1 diabetes management education and care can take place in different clinical settings. This study assessed metabolic outcomes (i.e. hemoglobin A1C), healthcare utilization and costs among new-onset type 1 diabetic children who received initial diabetes education and care in a hospital compared to those children in an outpatient pediatric endocrinology clinic.

**Methods:**

A retrospective cross-sectional study was conducted from the payer’s perspective. New-onset type 1 diabetic children, aged 1–18, presented at Baystate Children’s Hospital (Massachusetts) from 2008–2009 were included in the study if lab test confirmed diagnosis and there was one year of follow-up. Inpatients spent at least one night in the hospital during a 10-day diagnosis period and received all or part of diabetes education there. Outpatients were diagnosed and received all diabetes education in a pediatric endocrinology clinic. Metabolic outcomes were measured at diagnosis and at one year post-diagnosis. Healthcare charges and electronic medical records data were reviewed from 2008–2010. Healthcare costs components included diagnostic test, pediatric, endocrinology and hospitalists care, critical and emergency care, type 1 diabetes related supplies, prescription drugs, and IV products.

**Results:**

Study sample included 84 patients (33 inpatient and 51 outpatients). No statistically significant differences in patient demographic characteristics were found between groups. There were no statistically significant differences in metabolic outcomes between groups. Total cost at one year post-diagnosis per new-onset type 1 diabetic child was $12,332 and $5,053 in the inpatient and outpatient groups, respectively. The average healthcare cost for pediatric endocrinology care was $4,080 and $3,904 per child in the inpatient and outpatient groups, respectively.

**Conclusion:**

Provision of initial type 1 diabetes education and care to new-onset non-critically ill children in a hospital setting increases healthcare costs without improving patient’s glycemic control in the first year post-diagnosis.

## Background

Type 1diabetes mellitus (i.e. insulin-dependent diabetes mellitus) is one of the most prevalent chronic diseases among children in the United States [1]. In 2010, the prevalence of type 1 diabetes in children aged 0–19 in the United States was 1.7 per 1,000 children [2]. Among youth aged less than 10 years and 10–19 years there are 19.7 and 18.6 per 100,000 new cases of type 1 diabetes every year, respectively [1].

With proper education and care, type 1 diabetes can be managed throughout an individual’s life. Initial diabetes education is important for establishing successful diabetes self-management, long-term glycemic control, and complication free survival [3]. Often, new-onset type 1 diabetic children are admitted to hospitals for metabolic stabilization and disease education, regardless of disease severity [4,5]. While fifteen percent of newly diagnosed type 1 diabetic children require urgent insulin and fluid replacement treatment, one-third to one-half of them present with mild symptoms [6,7]. For children with mild to moderate symptoms and who are clinically stable, there is not definite evidence as to which healthcare setting is most cost-effective for the provision of initial type 1 diabetes management education and patient care [3]–[6,8]–[13].

Previous studies compared metabolic outcomes using subsequent hemoglobin A1C (i.e. HbA1C) levels of newly diagnosed type 1 diabetic children based on initial diabetes education setting [7]–[9,13,14]. Chase et al., (1992) found that HbA1C levels of newly diagnosed type 1 diabetic inpatient and outpatient children were not significantly different for any of the five follow-up years after diagnosis (p>0.05). Additionally, Simell et al. (1991) found that the length of initial stay in a hospital had no effect on metabolic control in newly diagnosed type 1 diabetic children at 2 years post- diagnosis. Dougherty, Soderstrom, & Schiffrin (1998) found that the mean HbA1C levels of the children, who received initial diabetes education in a home-based setting (6.4%; 46 mmol/mol), was not significantly different (p>0.55) from those in a hospital setting (6.1%; 43 mmol/mol) at 12 months post-diagnosis; nevertheless, HbA1C levels were significantly lower (p<0.05) in the home-based group (6.4%; 46 mmol/mol) than in the hospital group (7.1%; 54 mmol/mol) at 3 years after diagnosis. Similarly, Tiberg et al. (2012) did not find statistically significant differences (p>0.74) in the mean HbA1C levels at six months post-diagnosis of newly diagnosed type 1 diabetic children who received initial diabetes education in a home-based setting (6.0%; 42.8 mmol/mol) compared to children in a hospital setting (6.1%; 43.3 mmol/mol). Finally, Hamman et al. (1985) found no significant differences in the risk of subsequent ketoacidosis episodes among children who received their initial education in an inpatient, an outpatient, or a mixed inpatient/outpatient setting.

The United States ranked highest in the world for diabetes health expenditures per capita and as a percentage of total health expenditures [15]. The economic burden of type 1 diabetes in the United States was estimated at $14.9 billion in 2007, including $10.5 billion in healthcare costs [16]. The average annual type 1 diabetes healthcare cost per diabetic adult was estimated at $10,495 in 2007 [17]. Few studies have estimated the costs of type 1 diabetes diagnosis and care in new-onset children based on initial diabetes education setting [13,18]–[20]. Dougherty, Soderstrom, & Schiffrin (1998) estimated that the cost of type 1 diabetes diagnosis and care in children, who received their initial diabetes education in a home-based setting, was CAN$ 48 (USD 53) higher per child (p>0.85) compared to the inpatient setting. Similarly, Spaulding & Spaulding (1976) estimated that the total average cost per patient (both children and adults) in the home-based setting was CAN$ 154 (USD 169) compared to CAN$ 1,445 (USD 1,587) average total cost per patient admitted to the hospital. Authors used the Ontario Medical Association fee schedule to compare the healthcare cost of new-onset type 1 diabetic patients managed in a home-based setting, without admission to hospital during the 6 months of continuing insulin treatment, with those patients admitted to a hospital for initiation of insulin treatment. Healthcare costs included salaries of staff, physician’s fees, laboratory costs and daily hospital bed rate. The costs of insulin, other drugs and some of the home urine-testing supplies were not included in the analysis. More recently, Tiberg et al. (2012) examined the healthcare costs one month after diagnosis of newly diagnosed type 1 diabetic children who received hospital-based care versus hospital-based home care. Authors found that total mean healthcare cost per patient was lower in the hospital-based home care group (1,501 SEK; USD 235) compared to the hospital-based care group (2,143 SEK; USD 335) (p<0.001). Authors used the Swedish southern regional healthcare pricelist as a proxy for cost of healthcare services. Healthcare services included stay at hospital and hospital-based home *Family House*, outpatient visits, telephone consultations and healthcare professionals’ educational time. Simell, Simell, & Sintonen (1993) compared the cost of care of the length of initial hospital stay of children with newly diagnosed type 1 diabetes. The costs of care of a child with type 1 diabetes admitted to a hospital for 23±4 days totaled £10,834 (USD 7,333) compared to £6,928 (USD 4,689) per child admitted for 9±3 days (p<0.001). Authors concluded that shortening the initial hospital stay of children with newly diagnosed type 1 diabetes decreased total costs without influencing metabolic outcome during the first 2 years of disease.

Baystate Health’s type 1 diabetes management education and care is delivered through the Pediatric Endocrinology Division at Baystate Children’s Hospital and an outpatient pediatric endocrinology clinic. Initial type 1 diabetes management and patient education is provided during 2–4 hours for two consecutive days. The initial two days of diabetes education sessions are followed by daily nurse interaction with the family over the phone, when clinically needed, and a clinic follow up visit at 1 week, 1 month, 2 months, and quarterly thereafter. The same team of specialty nurses and a dietitian provides diabetes education and insulin management training to the parents and child and covers the same content regardless of education setting (i.e. hospital and/or outpatient pediatric endocrinology clinic). The role and involvement of the pediatric endocrinologist is also similar whether the child is an inpatient at Baystate Children’s Hospital or an outpatient at the outpatient pediatric endocrinology clinic. New-onset type 1 diabetes management and patient education is initiated to clinically stable patients, either in the outpatient pediatric endocrinology clinic or on the hospital ward floor of Baystate Children’s Hospital, within a 10-day period following diagnosis.

The healthcare cost and utilization of the provision of initial type 1 diabetes education and care in a hospital compared to an outpatient pediatric clinic has not been empirically estimated. This study sought to assess differences in outcomes and costs between new-onset type 1 diabetic children who received initial education and care in a hospital and in an outpatient pediatric endocrinology clinic. The study specifically compared: 1) HbA1C levels at diagnosis and one year post-diagnosis; and 2) healthcare utilization and costs associated with the provision of type 1 diabetes education and care during the first year from date of diagnosis in both healthcare settings.

## Methods

### Data sources

Baystate Health in Massachusetts served as the healthcare setting for this study. Baystate Health comprises several healthcare settings including Baystate Children’s Hospital, medical practices, and outpatient clinics [21]. Clinical data of all newly diagnosed type 1 diabetic children from 2008 to 2010 derived from the pediatric endocrine clinic database PEDRO Electronic Medical Record System (Pedrosoft LLC, Basking Ridge, NJ) and the electronic medical record database of Baystate Health’s Clinical Information System (Cerner Corporation, North Kansas City, MO). Economic data derived from two Baystate Health electronic financial accounting systems for all new-onset type 1 diabetic children in the study period. Databases did not include any patient identifiers. Databases were merged using a unique random number assigned to each patient in the study. Baystate Health Institutional Review Board granted study approval.

### Study design

**Figure 1:**
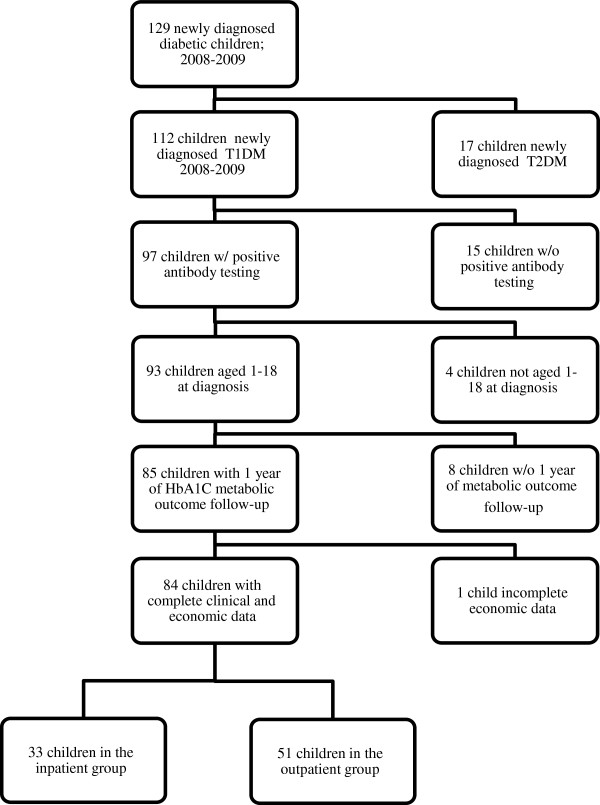
Flow diagram of the study population.

A retrospective cross-sectional study was conducted from the payer’s perspective. The study inclusion criteria of children were as follows: 1) newly diagnosed with type 1 diabetes evidenced by at least one positive antibody test for type 1 diabetes at diagnosis -i.e. islet cell antibody ICA512, islet cell antibody GAD65, and/or insulin antibody; 2) diagnosis occurred in the 2008–2009 period; 3) aged 1 to 18 years old at the time of diagnosis; and 4) metabolic outcome HbA1C data were available for at least one year of follow-up from diagnosis date. Children were excluded from the study if they did not have complete clinical and economic data. All children meeting inclusion criteria were divided into two groups; children were included in the inpatient group if they 1) spent at least one night in the hospital within the 10-day period after type 1 diabetes diagnosis, and 2) received all or part of the initial type 1 diabetes management education in the hospital. Children in the outpatient group were 1) diagnosed in an outpatient pediatric endocrinology clinic, and 2) received all initial diabetes management education there. A flow diagram of patient inclusion, exclusion, and group classification is shown in Figure 1.

Socio-demographic data included patient age, sex, race/ethnicity, health insurance type, and distance to Baystate Children’s Hospital. Race/ethnicity of patients was broken down into 4 categories: White Non-Hispanic, African-American, Hispanic, and other. Health insurance type was categorized either as public or private. Distance to hospital was defined as patient address to Baystate Children’s Hospital and as patient address within a 10 miles radius to Baystate Children’s Hospital.

HbA1C is the primary metabolic outcome used to measure patient’s glycemic control. This study followed the American Diabetes Association recommendation to adjust HbA1C levels by the age of the child based on: an HbA1C of less than 8.5% for children younger than age 6, 8.0% in children between ages 6 and 12, and under 7.5% for teenagers [22]. HbA1C levels were measured at diagnosis and one year post-diagnosis.

Economic data included healthcare charges for in-hospital healthcare services, intensive care, emergency care, and outpatient healthcare services. Charges were used as a proxy for healthcare cost estimations. Healthcare costs components included diagnostic tests (i.e. electrocardiograms, laboratory tests, and medical imaging tests), pediatric and endocrinology care, critical and emergency care, hospitalists care, and type 1 diabetes related supplies, prescription drugs, and IV products (i.e. IV solutions used for treatment). All charges were adjusted to 2011 dollars using the US city average and all items component of the consumer price index.

HbA1C levels and healthcare utilization and charges were compared among new-onset type 1 diabetic children based on clinical setting of initial type 1 diabetes education and care (i.e. inpatient or outpatient groups). Pediatric endocrinology care charges were broken down into its two components, type 1 diabetes follow-up care and diabetes management and patient education, to further assess type 1 diabetes cost differences between groups. Pediatric endocrinology care included physician consultation and visits (e.g. HbA1C, glucose, urinalysis testing; and other service related care). All type 1 diabetes follow-up care occurred either on the hospital ward floor or in the outpatient pediatric endocrinology clinic after patient initial diagnosis and clinical stabilization. Diabetes management and patient education included lifestyle counseling and education about diet, exercise, and insulin administration. Descriptive statistics were performed. Statistical differences using *t*-*test* and χ^2^ test were performed. Statistical significance was set at α=0.05.

## Results

### Descriptive statistics

The study included 84 patients; 33 received initial type 1 diabetes education and care at Baystate Children’s Hospital (i.e. inpatient group) and 51 in the outpatient pediatric endocrinology clinic (i.e. outpatient group). The percentage of female patients (63.63%), White non-Hispanics (75.76%), those with public health insurance (42.42%), and the average distance to the hospital (18.08 miles) were higher, but not statistically significant, in the inpatient group (Table 1). No statistically significant differences between groups were found in the socio-demographic characteristics of patients.

**Table 1:** Descriptive statistics

**Demographics**	**Inpatient**	**Outpatient**	**Total**
Patients (%)			
Patients by race/ethnicity	33 (39.28%)	51 (60.71%)	84 (100%)
White, Non-Hispanic	25 (75.76%)	38 (74.51%)	63 (75.00%)
African-American	2 (6.06%)	3 (5.88%)	5 (5.95%)
Hispanic	5 (15.15%)	8 (15.69%)	13 (15.48%)
Other	1 (3.03%)	2 (3.92%)	3 (3.57%)
% Female patients	21 (63.63%)	21 (41.18%)	42 (50%)
Age at diagnosis (Average±SD)	10.13±4.31	10.52±4.37	10.36±4.32
Distance to the hospital (in miles)	18.08±22.30	15.46±15.17	16.49±18.22
Patients living inside 10 miles to hospital	18 (54.55%)	29 (56.86%)	47 (55.95%)
Patients with public insurance	14 (42.42%)	16 (31.37%)	30 (35.71%)
**Healthcare services utilization**	**Inpatient**	**Outpatient**	**Total**
Hospital length of stay in days	1.84±0.75		0.72±1.02
Outpatient diabetes visits within 10 days of diagnosis date†	1.76±0.71	2.61±0.90	2.27±0.92
Inpatient diabetes visits within 10 days of diagnosis date†	0.97±0.39	0.02±0.14	0.39±0.54
Outpatient diabetes visits within 1 year of diagnosis date*	6.87±1.71	7.73±2.01	7.39±1.93
Phone calls received within 1 year of diagnosis date	14.39±8.68	11.02±8.15	12.35±8.48
**Clinical indicators for T1DM at diagnosis**	**Inpatient**	**Outpatient**	**Total**
Patients pH	29 (87.88%)	7 (13.73%)	36 (42.86%)
pH†	7.20±0.14	7.33±0.05	7.23±0.14
Patients Bicarb	33 (100%)	43 (84.31%)	76 (90.48%)
Bicarb†	14.94±7.81	23.98±3.92	20.05±7.41
Patients Blood Urea Nitrogen (BUN)	31 (93.94%)	42 (82.35%)	73 (86.90%)
BUN	16.94±8.53	14.24±3.94	15.38±6.40
**Metabolic outcome: HbA1C**	**Inpatient**	**Outpatient**	**Total**
Patients HbA1C at diagnosis	33 (100%)	51 (100%)	84 (100%)
HbA1C at diagnosis	11.60%±2.08%	10.70%±2.34%	11.05%±2.27%
Patients HbA1C 1 year post-diagnosis	33 (100%)	51 (100%)	`84 (100%)
HbA1c 1 year post-diagnosis	7.54%±1.56%	7.37%±0.89%	7.43%±1.20%
**Metabolic outcome: HbA1c adjusted by patient’s age**	**Inpatient**	**Outpatient**	**Total**
HbA1c at diagnosis	3.72±2.16	2.83±2.46	3.17±2.37
HbA1c 1 year post-diagnosis	−0.34±1.59	−0.50±0.88	−0.44±1.21
T1DM at diagnosis			
HbA1c ≤ 6.5%		1 (1.96%)	1 (1.19%)
HbA1c > 6.5%	33 (100%)	50 (98.04%)	83 (98.81%)
T1DM 1 year post-diagnosis diagnosis			
HbA1c ≤ 6.5%	8 (24.24%)	8 (15.69%)	16 (19.05%)
HbA1c > 6.5%	25 (75.76%)	43 (84.31%)	68 (80.95%)

Note: ±SD, Standard deviation. *, § and † indicates statistically significant at the 0.05, 0.01 and 0.001 levels, respectively. HbA1C levels at diagnosis in % and (mmol/mol units) are 11.6% (103 mmol/mol), 10.7% (93 mmol/mol) and 11.1% (97 mmol/mol) in the inpatient, outpatient and total group respectively. HbA1C levels at 1 year post diagnosis in % and (mmol/mol units) are 7.5% (58 mmol/mol), 7.4% (56 mmol/mol) and 7.4% (57 mmol/mol) in the inpatient, outpatient and total group respectively. Blood glucose levels higher than 11 mmol/L (i.e. hyperglycemia) with a venous pH below 7.3 and/or blood bicarbonate level below 15 mmol/L indicates pediatric diabetic ketoacidosis. Blood urea nitrogen (BUN) is normal in a range of 6–20 mg/dL. Pediatric endocrinology care visits include first visit.

The average hospital length of stay (ALOS) of the inpatient group was 1.84±0.75 days and the ALOS in the Intensive Care Unit (ICU) was 1.1±0.30 days. The outpatient group had a higher average number of pediatric endocrinology outpatient visits during the 10-day period following the initial diagnosis (2.61±0.90 outpatient group vs. 1.76±0.71 inpatient group; p<0.0001), and during the first year after diagnosis (7.73±2.01 outpatient group vs. 6.87±1.71 inpatient group; p=0.04). The average number of phone calls nurses received during the year of follow-up care was higher, but not statistically significant, in the inpatient group (14.39±8.68 inpatient group vs. 11.02±8.15 outpatient group).

At the time of diagnosis, the average blood pH (7.20±0.14 inpatient group vs. 7.33±0.05 outpatient group; p<0.001) and bicarbonate (14.94±7.81 vs. 23.98±3.92; p<0.001) levels were higher in the outpatient group (Table 1). The difference in BUN levels was not statistically significant.

### Health outcomes

Study results showed no statistically significant differences between groups in the average HbA1C levels at diagnosis (11.60%±2.08% inpatient group vs. 10.70%±2.34% outpatient group) and at one year post-diagnosis (7.54%±1.56% vs. 7.37%±0.89%). HbA1C levels at diagnosis were 3.72 and 2.83 percentage points above HbA1C target levels based on the child age in the inpatient and outpatient groups respectively (p=0.08); and 0.34 and 0.50 percentage points, respectively below HbA1C target levels at one year post-diagnosis (p= 0.58).

### Healthcare services utilization and costs by clinical setting

Overall, children in the inpatient group used more healthcare services than those in the outpatient group during the first year post-diagnosis (Table 2). The average total utilization of healthcare services per child was 2.6 times higher among children in the inpatient group than in the outpatient group (156.24±68.67 inpatient groups vs. 58.67±15.35 outpatient group, p<0.001); and the average total charges per child were 2.4 times higher in the inpatient group ($12,332±$4,251 vs. $5,053±$1,469, p<0.001). The average utilization (68.45±28.46 and 11.92±7.80, respectively, p<0.001) and charges ($4,210±$1,565 and $882±$639, respectively, p<0.001) per child for hospital care (excluding emergency and intensive care) were respectively 5.7 and 4.7 times higher among children in the inpatient group than in the outpatient group; whereas, the average utilization and charges per child for outpatient care were higher, although not statistically significant, among children in the outpatient group. The main cost drivers in the inpatient group were hospital care (34.14% of total inpatient charges), outpatient care (29.47%), intensive care (26.47%), and emergency care (9.93%). Likewise, the main cost drivers in the outpatient group were outpatient care (78.85% of total charges), hospital care (17.46%), and emergency care (3.69%).

**Table 2:** **Type 1 diabetes healthcare utilization and charges by level of care**, **2008**–**2010**

	**Emergency care**	**Hospital care**	**Intensive care**	**Outpatient care**	**Total**
**Total sample**					
# Patients	32	73	20	84	84
Average±SD utilization per patient	1.33±2.33	34.13±33.47	17.98±39.51	43.54±15.34	97±65.25
95% CI Lower-Upper utilization	0-3.18	22.48-45.77	12.34-23.63	21.51-65.59	120.58-156.24
Average±SD charges per patient	$594.06±$980.40	$2,189.67± $1,965.53	$1,282.26± $2,610.84	$3,846.59± $1,304.85	$7,912.58± $4,588.84
95% CI Lower-Upper charges	$555.99-$632.12	$2,090.61-$2,288.73	$1,231.93-$1,332.58	$3,633.01-$4,060.17	$7,678.36-$8,146.81
**Inpatient group**					
# Patients	22	33	20	33	33
Average±SD utilization per patient	2.55 ± 2.83*	68.45 ± 28.46*	45.79 ± 52.32*	39.45±15.56	156.24±68.67
95% CI Lower-Upper utilization	0-6.07	43.10-93.81	33.34-58.23	19.49-59.43	119.11-193.38
Average±SD charges per patient	$1,224.09±$1,190.47*	$4,210.16±$1,564.92*	$3,263.94± $3,318.15*	$3,633.64± $1,258.32	$12,331.83±$4,251.35*
95% CI Lower-Upper charges	$1,152.94-$1,295.24	$3,996.75-$4,423.58	$3,150.32-$3,377.56	$3,428.22-$3,839.06	$11,952.55-$12,711.12
**Outpatient group**					
# Patients	10	40		51	51
Average±SD utilization per patient	0.55±1.51	11.92±7.80		46.20±14.76	58.67±15.35
95% CI Lower-Upper utilization	0-1.63	3.09-20.75		22.00-70.39	28.98-88.36
Average±SD charges per patient	$186.38±$506.98	$882.29±$638.69		$3,984.39±$1,328.05	$5,053.07±$1,469.05
95% CI Lower-Upper charges	$169.78-$202.57	$812.28-$952.31		$0.00-$4,203.70	$4,788.66-$5,317.48

Note: ±SD, Standard deviation; CI, Confidence Interval. * Indicates statistically significant p< 0.001. Utilization in units of healthcare services. Adjusted to 2011 US dollars.

Healthcare utilization in the inpatient group was 5 times higher for diagnostic tests (p<0.001) and 1.5 to 2.3 times higher for outpatient pediatric care and pediatric hospitalists care, respectively (Table 3). Utilization of medical supplies (p=0.05) and pharmaceuticals (p<0.001) was also higher among children in the inpatient group. There were significant differences in average charges per patient for the same services provided to children in the inpatient group and those in the outpatient group (Table 3). Average charges per child for diagnostic test (p<0.001), hospital pediatric care (p<0.001), hospitalist care (p<0.001) and emergency care (p<0.001) were significantly higher for children in the inpatient group than those in the outpatient group. Average charges per child for medical supplies (p<0.001) and pharmaceuticals (p=0.05) were also higher for children in the inpatient group. Pediatric endocrinology care and diagnostic tests represented 77.26% and 15.60%, respectively of the total cost for children in the outpatient group. Likewise, the main cost drivers for children in the inpatient group were pediatric endocrinology care (33.08% of total cost for children in the inpatient group) followed by diagnostic tests (18.88%) and pediatric critical care (17.16%).

**Table 3:** **Type 1 diabetes cost components by group**, **2008**–**2010**

	**Diagnostic test**	**Emergency care**	**Hospital pediatric care**	**Pediatric critical care**	**Pediatric endocrinology care**	**Pediatric cardiology care**	**Pediatric hospitalist care**	**Outpatient pediatric care**	**Medical supplies**	**Prescription drugs**	**IV products**	**Total**
**Total sample**												
# Patients	80		7	20	84	14	25	6	32	38	37	84
Average±SD utilization per patient	34.65 ± 36.64	3.5 ± 2.59	3.57 ± 6.55	1.44 ± 3.03	43.05 ± 15.25	1.29 ± 0.61	2.24 ± 1.76	3.33 ± 3.83	2.75 ± 2.09	21.71 ± 26.75	5.38 ± 3.97	97.00 ± 65.25
95% CI Lower- Upper utilization	23.33-45.97	0-8.35	0.71-6.43	0-3.07	21.26-64.83		0-6.63	0-7.11	0-6.56	13.37-30.06	0-11.46	73.42-120.58
Average±SD charges per patient	$1,393.26±$1,119.60	$594.06±$980.40	$857.29±$1,231.62	$831.52±$1,656.26	$3,972.91±$1,300.76	$6.42±$28.57	$123.20±$219.48	$26.51 ± $139.96	$6.91±$69.80	$8.08±$37.15	$12.66±$21.86	$7,912.58±$4,588.84
95% CI Lower- Upper charges	$1,309.74-$1,476.78	$555.99-$632.12	$808.29-$906.29	$790.54-$872.49	$3,751.96-$4,193.86	$4.00-$8.83	$106.50-$139.90	$22.10 - $30.91	$20.42-$33.41	$5.40-$10.76	$7.12-$18.21	$7,678.36-$8,146.48
**Inpatient group**												
# Patients	33	22	3	20	33	10	24	3	28	33	32	33
Average±SD utilization per patient	65.64 ± 39.84*	3.82 ± 2.87	7.85 ± 8.39*	3.67 ± 3.93*	40.76 ± 15.92	1.30 ± 0.67	2.29 ± 1.78	4.00 ± 5.20	2.86 ± 2.22^§^	24.76 ± 27.47*	5.94 ± 3.96*	156.24 ± 68.67*
95% CI Lower- Upper utilization	45.04-86.24	0-9.11	2.41-13.29	0-7.82	20.13-61.38	0-6.78	0.49-7.51	0-6.82	0-6.82	15.42-34.10	0-12.66	119.11-193.38
Average±SD charges per patient	$2,328.85±$1,197.98*	$1,224.09±$1,190.4*	$2,035.76±$1,192.99*	$2,116.57±$2,076.10*	$4,079.84±$1,300.87	$6.66±$10.72	$250.05±$253.98*	$39.68±$188.80	$64.07±$100.50*	$20.58±$57.57§	$31.33±$25.32*	$12,331.83±$4,251.35*
95% CI Lower- Upper charges	$2,193.90-$2,463.80	$1,152.94-$1,295.24	$1917.54-$2,153.99	$2,023.41-$2,209.73	$3,852.94-$4,306.74	$2.53-$10.79	$259.71-$334.58	$33.88-$45.48	$51.26-$76.89	$15.13-$26.01	$18.79-$43.85	$11,952.55-$12,711.12
**Outpatient group**												
# Patients	47	10	4		51	4	1	3	4	5	5	51
Average±SD utilization per patient	12.89 ± 5.31	2.8 ± 2.39	0.80 ± 2.51		44.53 ± 14.76	1.25 ± 0.50	1 ±0	2.67 ± 2.89	2 ± 0	1.6 ±0.89	1.8 ± 1.30	58.67 ± 15.35
95% CI Lower- Upper utilization	1.59-24.20	0-6.68	0.31-1.92		21.20-67.85			0-6.36			0-5.33	28.98-88.36
Average±SD charges per patient	$787.87±$459.07	$186.38±$506.98	$94.75±$312.19		$3,903.72±$1,308.90	$6.26±$35.80	$10.65±$76.06	$20.02±$112.49	$2.87±$11.03	$	$0.60±$2.14	$5,053.07±$1,469.05
95% CI Lower- Upper charges	$714.09-$861.66	$169.78-$202.99	$83.99-$105.51		$3,687.31-$4,120.12	$4.16-$8.36	$8.20-$13.11	$16.22-$23.81	$1.09-$4.64	$	$(0.00)-$1.41	$4,788.66-$5,317.48

Note: ±SD, Standard deviation; CI, Confidence Interval. * and § indicates statistically significant at 0.001 and 0.05 levels, respectively. Utilization is in units of healthcare services. Healthcare services used by a total 5 or less patients are not shown in Table. Adjusted to 2011 US dollars.

The average total charges per type 1 diabetic patient for pediatric endocrinology care were higher for children in the inpatient group (Table 4). The average charges per patient for follow-up diabetes care were higher for patients in the inpatient group ($2,436±$567 inpatient group vs. $2,092±651 outpatient group, p=0.01); whereas, the average charges per patient for diabetes management education were higher, but not statistically significant, for patients in the outpatient group ($1,812±$1,113 vs. $1,643±$1,129).

**Table 4:** **Pediatric endocrinology charges by group**, **2008**–**2010**

	**Follow**-**up diabetes care**	**Diabetes management education**
**Total sample**		
# Patients	84	84
Average±SD charges per patient	$2,227.22±$638.57	$1,745.68±$1,115.90
Min. charges per patient	$614.36	$115.13
Max. charges per patient	$3,356.5	$4,657.45
**Inpatient group**		
# Patients	33	33
Average±SD charges per patient	$2,436.49±$567.14^§^	$1,643.35±$1,129.25
Min. charges per patient	$702.28	$115.13
Max. charges per patient	$3,338.71	$4,588.37
**Outpatient group**		
# Patients	51	51
Average±SD charges per patient	$2,091.82±$650.72	$1,811.90±$1,113.36
Min. charges per patient	$614.36	$172.69
Max. charges per patient	$ 3,356.5	$4,657.45

Note: ±SD, Standard deviation. § Indicates statistically significant p=0.01; Adjusted to 2011 US dollars.

## Discussion

Often, children with new-onset type 1 diabetes who present with acute symptoms of diabetes and markedly elevated blood glucose levels are admitted to a hospital [4]–[6]. However, evidence from previous studies suggests that for non-critically ill type 1 diabetic patients, insulin therapy initiated in the hospital does not yield improved metabolic outcomes when compared to diabetes care initiated in an outpatient setting [9,11,12,23]. In the late 1980s, some pioneer experiences shifting to outpatient type 1 diabetes treatment showed the potential for decreasing diabetes-related hospital admissions, length of stay and healthcare costs [10]. In the current environment of healthcare reform and heightened awareness of the cost of healthcare, there is renewed interest in understanding differences in outcomes and cost of type 1 diabetes based on clinical setting of initial diabetes education and care.

This study advances the analysis of differences in the cost of care provided to patients in different clinical settings. While costly provision of care in United States hospitals have been documented elsewhere, novel findings of this study showed significant differences between clinical settings in the average charges per patient for the provision of the *same* healthcare services including diagnostic tests, hospital pediatric care, hospitalist care, medical supplies, and pharmaceuticals.

Furthermore, study results showed that average charges per patient for type 1 diabetes follow-up care provided on the hospital ward floor were higher than in the outpatient pediatric endocrinology clinic. The average total charges per new-onset type 1 diabetic patient for pediatric endocrinology care, including follow-up diabetes care and diabetes management and patient education, was $4,080 per child in the inpatient group compared to $3,904 per child in the outpatient group. All type 1 diabetes follow-up care at Baystate Health occurred after initial diagnosis period when the patient was clinically stable and thus, differences in pediatric endocrinology charges between clinical settings are not related with the patient’s diabetes control or severity of condition at diagnosis.

## Limitations

Some limitations must be taken into account in the interpretation of the study results. There is a potential for selection bias, which is inherent to any non-randomized study. As a result, there were sicker patients within the inpatient group, as evidenced by laboratory test results at diagnosis, who by the virtue of their condition had a need for more medical services resulting in higher costs. Nevertheless, new-onset type 1 diabetes management and patient education is initiated, either in the outpatient pediatric endocrinology clinic or on the hospital ward floor of Baystate Children’s Hospital, only when the patient is clinically stable within a 10-day period following diagnosis. Thus, differences in healthcare services utilization and costs of pediatric endocrinology care, diabetes follow-up care, and diabetes education and management training between clinical settings are not related with the patient’s diabetes control or severity of condition at diagnosis.

All new-onset type 1 diabetic children presenting at Baystate Health from 2008–2009, who had metabolic HbA1C outcome data available for at least one year of follow up from the date of diagnosis, were candidates for inclusion in the study. Still, the study sample size could impact the results.

The inherent variability in medical practice may also impact study results. Blood pH, bicarbonate, and BUN test results were used to evaluate a patient’s clinical stability and diabetes condition severity at diagnosis. The statistically significant differences observed in these laboratory tests at diagnosis could be related with the number of patients tested for clinical indicators of the disease. Also, many factors, besides clinical stability at diagnosis, contribute to the decision as to whether or not a child is hospitalized at the diagnosis of diabetes; physicians may weigh a patient’s specific circumstances, such as the family’s socioeconomic status or distance from the medical institution, and setting-specific factors, such as number of pediatric diabetes clinicians and diabetes educators at the institution, when choosing hospital admission as the site of initial type 1 diabetes management and education.

One year of follow-up data was used in this study. Additional years of follow-up data would have provided more accurate clinical evidence of the difference in the long-term metabolic control of newly diagnosed type 1 diabetic children based on the initial clinical setting of diabetes education. Furthermore, HbA1C levels at one year post-diagnosis could reflect, in part, a temporary disease remission following initial care called the *honeymoon period*[24].

Lastly, direct healthcare costs estimations are based on charges to third-party payers. Average charges per type 1 diabetic patient for healthcare services could overestimate the cost of the provision of diabetes education and care to the provider. Also, actual health insurance reimbursement rates for healthcare services were unknown to the researchers. Health insurance companies may reimburse healthcare providers at different rates for the same service, and some insurers may have pre-negotiated allowable charges for particular services. There is also a potential for incomplete patient service charges data related to type 1 diabetes. All data in this study were gathered for services associated with type 1 diabetes and provided within the Baystate Health network. Any services related to type 1 diabetes care provided outside of the Baystate Health system are unknown to the researchers.

## Conclusion

Hospital admission for type 1 diabetes initial care, patient education and insulin management training in new-onset non-critically ill children did not result in better metabolic outcomes during the first year post-diagnosis and increased cost. Thus, this study provides empirical evidence to support a shift from the current practice of hospital admission to outpatient provision of care in type 1 diabetic non-critically ill children for initial diabetes education and care.

## Competing interests

The authors declare that they have no competing interests.

## Authors’ contributions

RRM designed, conducted and directed this study. CFJ collaborated in the conceptualization and design of the study under RRM supervision and extracted the data. RRM and CFJ managed the data, conducted the statistical analysis, and wrote the manuscript. HA and KT conceived the idea of conducting this study, wrote the original protocol, and supervised the overall IRB process. HA and KT reviewed the manuscript and contributed to discussion. All authors read and approved the final version for the manuscript.

## Pre-publication history

The pre-publication history for this paper can be accessed here:

http://www.biomedcentral.com/1471-2431/13/55/prepub
